# Determining the safety of ultrafocal salvage high-dose-rate brachytherapy for radiorecurrent prostate cancer: A toxicity assessment of 150 patients

**DOI:** 10.1016/j.ctro.2020.12.002

**Published:** 2020-12-11

**Authors:** Marieke van Son, Max Peters, Marinus Moerland, Sandrine van de Pol, Wietse Eppinga, Jan Lagendijk, Jochem van der Voort van Zyp

**Affiliations:** Department of Radiotherapy, University Medical Center Utrecht, Heidelberglaan 100, 3584 CX Utrecht, the Netherlands

**Keywords:** Toxicity, CTCAE 4.0, Prostate cancer, Local recurrence, Focal salvage therapy

## Abstract

**Background and purpose:**

Local re-treatment of radiorecurrent prostate cancer is potentially curative. However, the increased risk of severe toxicity may outweigh the opportunity of cancer control. This study aims to evaluate treatment-related toxicity from ultrafocal salvage high-dose-rate brachytherapy (HDR-BT) and to investigate potential risk factors.

**Materials and methods:**

Toxicity data from 150 treated patients (July 2013–November 2019) was collected from a prospective registry. The treatment aim was to deliver a single dose of 19 Gy to the recurrent lesion as identified on multiparametric MRI and PET-CT. Treating physicians graded genitourinary (GU) and gastro-intestinal (GI) toxicity and erectile dysfunction (ED) using the Common Terminology Criteria for Adverse Events (CTCAE) 4.0, at baseline and during follow-up. Domains with substantial (≥10%) new-onset grade ≥ 2 toxicity were further evaluated using mixed effects logistic regression to find potential risk factors.

**Results:**

Median follow-up time was 20 months (IQR 12–31). Over time, new-onset grade 2 and 3 toxicity was recorded in 41% and 3% (GU), 5% and 0% (GI) and 22% and 15% (ED). While GI toxicity remained stably low, grade ≥ 2 GU toxicity and ED were seen twice as frequent in the late phase (>3 months after treatment). Significant risk factors for grade ≥ 2 toxicity were baseline GU toxicity (grade ≥ 2), baseline ED (grade ≥ 2), IPSS (cut-off ≥ 14) and urethral dose (D10%, cut-off ≥ 17 Gy).

**Conclusion:**

Ultrafocal salvage HDR-BT is a safe re-treatment option, especially in patients with a favorable symptom profile at baseline. Adherence to urethral dose constraints is important to avoid GU toxicity.

## Highlights

• Severe toxicity is as low as 3% (GU), 0% (GI) and 15% (ED).
• Lower impact is predicted for patients with favorable function at baseline.
• Lower dose to the urethra (D10% <17 Gy) may prevent urinary symptoms.

## 1 Introduction

Patients with a local prostate cancer recurrence after radiotherapy are potential candidates for curative salvage treatment, which offers the opportunity to avoid or postpone palliative androgen deprivation therapy (ADT), thereby preventing patients from its associated metabolic, cardiovascular, sexual and psychological side-effects [1], [2]. Whole-gland salvage treatments are generally associated with (severe) side-effects. A recent prospective study on whole-gland salvage brachytherapy reported 14% grade 3 toxicity [3]. The aim of focal treatment is to solely target the recurrent tumor and therefore further reduce toxicity, potentially with comparable cancer control.

Improvements in imaging for selection and treatment, most notably prostate specific membrane antigen (PSMA)-PET/CT, have advanced the field of focal salvage treatment [4]. Across different modalities, toxicity from focal salvage treatment seems limited compared to whole-gland salvage treatment, with event rates of severe (grade 3) genitourinary (GU) and gastro-intestinal (GI) toxicity as low as 5% and erectile dysfunction (ED) often reduced, allowing some patients to preserve their potency [5], [6], [7].

However, reported series in literature are mostly retrospective and small, using a wide range of patient- and physician-reported toxicity outcome measures. This leads to bias and prevents adequate assessment of risk factors which could be used to reduce or avoid associated side-effects of treatment.

We previously reported tumor control and functional outcomes of 50 patients after two years follow-up [8] and we investigated patient-reported quality of life of 100 patients treated with MRI-guided ultrafocal salvage high-dose-rate brachytherapy (HDR-BT) [9]. With an emphasis on further safety evaluation, the current study reports prospectively collected data of physician-graded GU and GI toxicity and ED in a total of 150 treated patients. Additionally, we analyze potential risk factors for toxicity to improve treatment planning and to guide patient counselling.

## 2 Materials and methods

### 2.1 Patients

We used data from a single-center prospective registry of patients treated with ultrafocal salvage HDR-BT. The first consecutive 150 patients were included, treated between July 2013 and November 2019. As described previously [9], patients were either treated within an institutional review board (IRB)-approved study (Netherlands Trial Register 6123 or 7014) or outside the scope of a study protocol if tumor characteristics were incompatible with study inclusion criteria. All patients (on- or off-protocol) were prospectively followed in the same manner. Pre-treatment characteristics varied from lower- to higher-risk disease, but acceptable baseline urinary toxicity (International Prostate Symptom Score [IPSS] < 15) was required for all patients. Study patients all signed informed consent. The IRB waived the requirement for informed consent for off-protocol patients.

### 2.2 Intervention

Before treatment, patients underwent 3T multiparametric (mp)-MRI (T2-weighted, diffusion-weighted and dynamic contrast enhanced imaging) without an endorectal coil and 68 Ga-PSMA or Choline PET-CT. Both imaging modalities were used to delineate the gross tumor volume (GTV), clinical target volume (CTV, defined as five-millimeter margin around GTV, excluding the urethra) and organs at risk (OARs: bladder, rectum, and urethra). Suspicious areas on MRI- or PET-imaging were included in the GTV, even if exclusively present on one of them. Patients treated before 2018 also underwent systematic (21/150) or (systematic and) MRI-targeted biopsies (67/150). After that, patients were treated without biopsy confirmation. Treatment was performed by trans-perineal insertion of MR-compatible catheters in and around the CTV, with the patient under spinal anesthesia. Rigidly fused MRI/transrectal ultrasound images offered image guidance [10]. After the implantation a subsequent 1.5T MRI scan was used for delineation adjustment and catheter reconstruction. The goal was to deliver 1 × 19 Gy to the CTV (D95%), with dosimetry constraints for the bladder and rectum (D1cc < 12 Gy) and for the urethra (D10% <17.7 Gy). Since radiation is fully targeted at the CTV (and not a quarter or half of the gland), this treatment is generally described as ultrafocal.

### 2.3 Outcome assessment

Toxicity before and after treatment was graded by the treating physician using the Common Terminology Criteria for Adverse Events (CTCAE) 4.0. Prostate cancer-specific domains were GU toxicity (6 subdomains), GI toxicity (10 subdomains) and ED. Each domain/subdomain was graded according to the severity of the symptoms, with a general range from grade 1 (asymptomatic or mild) to grade 2 (moderate), grade 3 (severe but not immediately life-threatening), grade 4 (life-threatening) and grade 5 (death). Toxicity grading was performed at baseline and during follow-up visits after 1, 3, 6, 9, 12, 18 and 24 months, and yearly thereafter.

### 2.4 Statistical analysis

To assess the effect of ultrafocal salvage HDR-BT on toxicity, post-treatment toxicity grades were compared to baseline grade. Any score above baseline was considered new-onset toxicity and therefore potentially treatment-related. The overall grade for the domains GU and GI toxicity was determined by the highest score of the respective subdomains.

For the domains showing substantial (≥10%) new-onset grade ≥ 2 toxicity, an explorative risk factor assessment was performed to study the effect of (pre)-treatment characteristics. Potential risk factors included patient-reported baseline symptoms (IPSS and IIEF-5) and physician-graded baseline toxicity (CTCAE 4.0), dose to the respective OAR, stage/location of the tumor, prostate size, CTV size, primary treatment type, history of previous salvage treatment, interval between primary and current salvage treatment, history of transurethral resection of the prostate (TURP) or ADT and number of brachytherapy catheters used. Using the lme4 package [11], mixed effects logistic regression was performed to model development of grade ≥ 2 toxicity over time, with potential risk factors included as fixed effects and a random effect per patient and per follow-up time point. In this multivariable model, odds ratios and their 95% confidence intervals (CI) were calculated to assess the independent effect of each risk factor on the outcome, with p-values < 0.05 considered statistically significant.

Statistical analyses were performed with R statistical software (version 3.5.1; the R foundation for Statistical Computing, Vienna, Austria) and IBM SPSS statistics (version 23.0).

## 3 Results

Table 1 summarizes baseline characteristics. Most patients were primarily treated with EBRT or low-dose-rate (LDR)-BT, with 20% receiving (neo)-adjuvant ADT in the primary setting. A small group (<5%) had already received a previous salvage treatment. Median interval between primary treatment and current salvage treatment was 8 years. Seven patients presented with a solitary lymph node or bone metastasis for which they received upfront stereotactic radiotherapy. Baseline GU and GI toxicity was limited to 12% and < 2% grade 2 toxicity, respectively. Approximately half of all patients had grade ≥ 2 ED at baseline. Dosimetry constraints were adhered to in 83% of patients, with maximum outliers to 18.5 Gy (urethra D10%), 14.5 Gy (bladder D1cc) and 12.6 Gy (rectum D1cc). Median follow-up time was 20 months (IQR 12–31).

**Table 1:** Patient and treatment characteristics (n = 150).

			Median (IQR) or number (%)	Missing (%)
Age (years)			72 (68–75)	
Primary treatment	EBRT		80 (53.3%)	
	LDR-BT		67 (44.7%)	
	Whole-gland HDR-BT		2 (1.3%)	
	Ultrafocal HDR-BT		1 (0.7%)	
History of ADT*	No		120 (80%)	
	Neo-adjuvant		8 (5.3%)	
	Adjuvant		22 (14.7%)	
Previous salvage treatment	No		143 (95.3%)	
	Whole-gland LDR-BT		4 (2.7%)	
	Ultrafocal HDR-BT		3 (2%)	
History of TURP			10 (6.7%)	
Interval primary–salvage treatment (years)			8 (5.3–10.7)	
TNM-stage on imaging	T	T2	85 (56.7%)	
		T3	63 (42%)	
		T4	2 (1.3%)	
	N	N0	146 (97.3%)	
		N1	4 (2.7%)	
	M	M0	147 (98%)	
		M1	3 (2%)	
Dorsolateral location of the tumor			132 (88%)	
Size of the CTV (cc)			8.5 (6–12.8)	
Size of the prostate# (cc)			31.4 (25.7–39.6)	
Baseline IPSS			8 (5–11)	16 (10.7%)
Baseline IIEF			9 (4–18)	24 (16%)
Baseline toxicity^	GU	0	73 (48.7%)	6 (4%)
		1	52 (34.7%)	
		2	19 (12.6%)	
		3	0 (0%)	
	GI	0	118 (78.7%)	6 (4%)
		1	24 (16%)	
		2	2 (1.3%)	
		3	0 (0%)	
	ED	0	23 (15.3%)	6 (4%)
		1	40 (26.7%)	
		2	54 (36%)	
		3	27 (18%)	
Number of brachytherapy catheters used			9 (8–11)	
CTV D95%			18.8 (17.4–19.7)	
Urethra D10% (Gy)			15.2 (10.3–17.5)	
Bladder D1cc (Gy)			9.2 (5.2–11.6)	
Rectum D1cc (Gy)			10.2 (8.1–11.7)	

Legend: IQR: interquartile range, EBRT: external beam radiotherapy, LDR-BT: low-dose-rate brachytherapy, HDR-BT: high-dose-rate brachytherapy, ADT: androgen deprivation therapy, TURP: transurethral resection of the prostate, TNM-stage: tumor/node/metastasis stage, CTV: clinical target volume, IPSS: international prostate symptoms score, IIEF: international index of erectile function.

* As part of primary treatment.

^ As graded by the Common Terminology Criteria for Adverse Events (CTCAE) 4.0.

# As measured on MRI.

### 3.1 Cumulative toxicity

Over time, 48/150 (32%) patients had maximum new-onset grade 1 GU toxicity, mainly consisting of mild urinary tract pain, hematuria or frequency. A maximum of grade 2 GU toxicity was seen in 61/150 patients (41%), mostly within the subdomain urinary frequency (49/61), for which medication was usually prescribed. Five patients (3%) experienced grade 3 GU toxicity. One patient had grade 3 cystitis, for which he received intravenous antibiotics during a hospital admission. Two patients had grade 3 urinary retention (urethral stricture), which involved placement of a permanent suprapubic catheter after failed urethral stricture incision. Two patients had grade 3 urinary incontinence: one had overflow incontinence due to bladder neck stenosis and one had severe stress incontinence.

Highest recorded new-onset GI toxicity was grade 1 in 47/150 patients (31%), which was mainly mild flatulence, rectal discomfort or mild rectal hemorrhage. Maximum grade 2 GI toxicity occurred in 8/150 patients (5%), mainly in the form of rectal hemorrhage needing minor cauterization (4/8). No grade 3 GI toxicity was seen.

In 7/150 (5%) patients, highest recorded new ED was grade 1. Maximum grade 2 ED was seen in 33/150 patients (22%) and maximum grade 3 ED in 22/150 (15%) patients.

### 3.2 Toxicity per time point

**Fig. 1:**
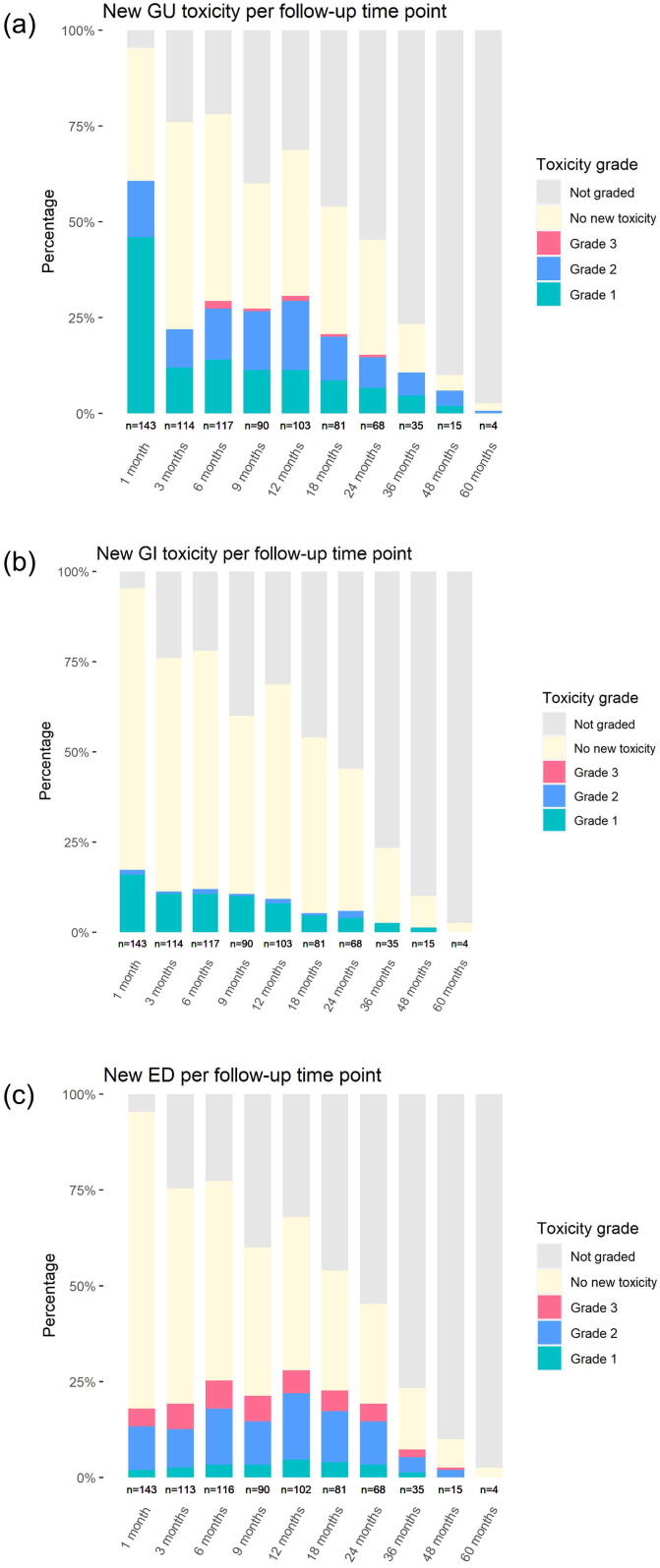
a–c: Stacked barplots displaying number of patients with new-onset toxicity after ultrafocal salvage HDR-BT. At each follow-up time point, toxicity scores were compared with baseline. Any score above baseline was considered new-onset toxicity.

A subdivision of new-onset toxicity per follow-up time point is graphically displayed in Fig. 1a–c. At each time point, the bars represent toxicity as compared to baseline. New grade 1 GU toxicity was mostly recorded in the first month, while grade 2 and 3 GU toxicity peaked between six and twelve months. GI toxicity generally decreased over time. The occurrence of grade 2–3 ED was relatively stable. For more detail, Supplementary Figs. 1a–f and 2a–j display new-onset toxicity per GU/GI subdomain.

### 3.3 Acute/late phase

Table 2 shows new-onset toxicity as divided into the acute (≤3 months) and late (>3 months) phase. For the GU domain, grade 2 toxicity increased from 21% (acute phase) to 41% (late phase). Grade 3 GU toxicity only occurred in the late phase. Grade 2 GI toxicity was limited to 2% and 5% in the acute and late phase, respectively. Grade ≥ 2 ED increased from 22% in the acute phase to 40% in the late phase. Supplementary Table 1 displays acute and late new-onset toxicity for each subdomain.

**Table 2:** New-onset acute and late toxicity.

Domain	Acute, number (%)	Missing, number	Late, number (%)	Missing, number
*Genitourinary toxicity*				
No toxicity	48 (33.4%)	6	46 (36.2%)	23
Grade 1	66 (45.8%)		24 (18.9%)	
Grade 2	30 (20.8%)		52 (40.9%)	
Grade 3	0 (0%)		5 (3.9%)	

*Gastrointestinal toxicity*				
No toxicity	112 (77.8%)	6	89 (70.1%)	23
Grade 1	29 (20.1%)		32 (25.2%)	
Grade 2	3 (2.1%)		6 (4.7%)	
Grade 3	0 (0%)		0 (0%)	

*Erectile dysfunction*				
No toxicity	108 (75%)	6	69 (54.8%)	24
Grade 1	4 (2.8%)		6 (4.8%)	
Grade 2	21 (14.6%)		32 (25.4%)	
Grade 3	11 (7.6%)		19 (15%)	

Legend: New-onset toxicity after ultrafocal salvage HDR-BT as graded by the Common Terminology Criteria for Adverse Events (CTCAE) 4.0. Any score above baseline in the acute (≤3 months) or late (>3 months) phase was considered new-onset toxicity.

**Fig. 2:**
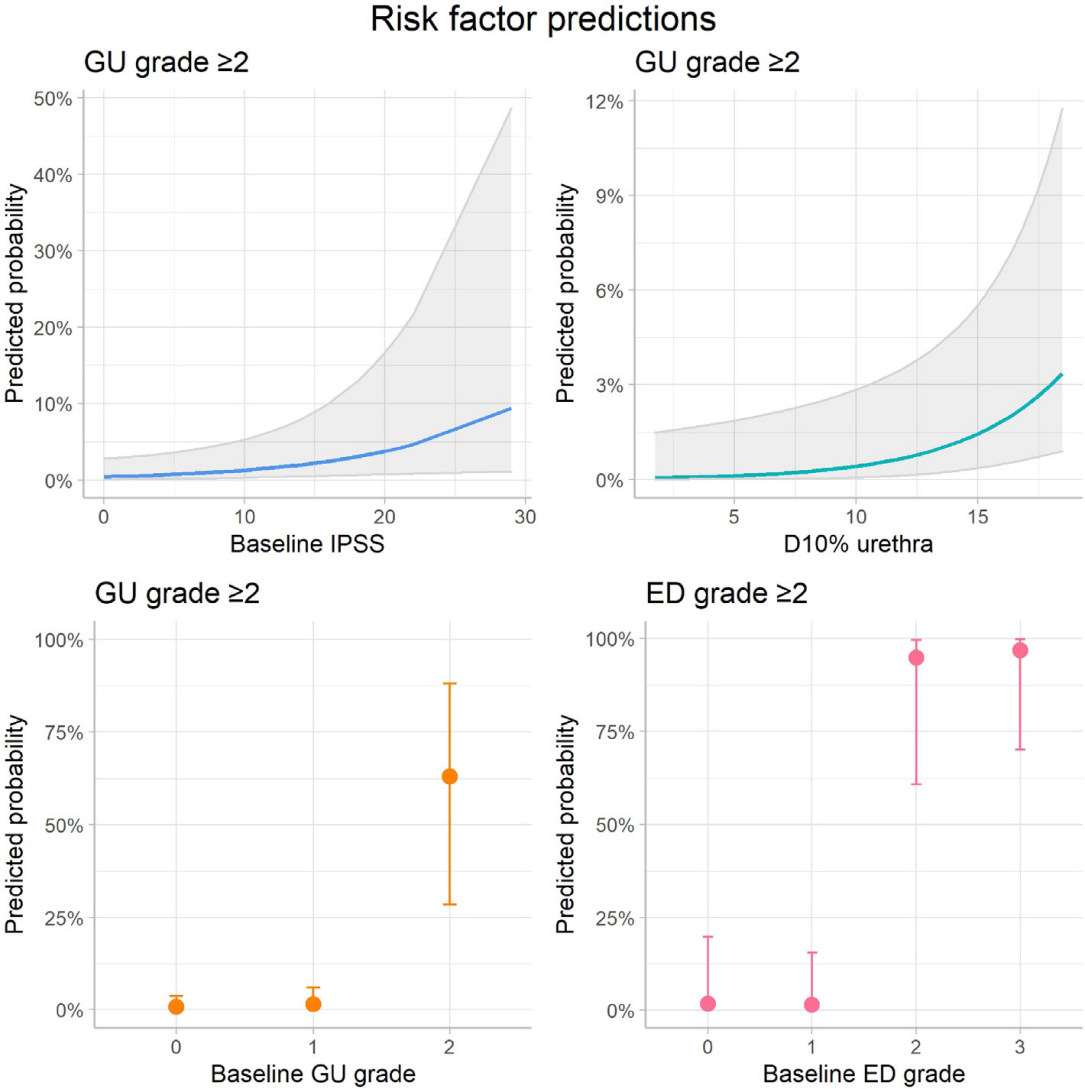
Predicted probabilities of grade ≥ 2 GU toxicity or ED at various levels of each risk factor. Modelled marginal means and their confidence intervals are shown, holding the other variables in the model constant.

For the domains GU toxicity and ED, an explorative risk factor assessment was performed (Table 3). In both domains, baseline toxicity appeared to be the strongest predictor of grade ≥ 2 toxicity (GU OR 14.8; ED OR 73.7). Within the GU domain, higher baseline IPSS (OR 1.11) and higher dose to the urethra (D10%) (OR 1.28) were also significant risk factors. Post-hoc cut-off analyses showed that the lowest contributive values to the model were IPSS ≥ 14 and urethra D10% ≥17 Gy. A baseline toxicity score of grade 2 or higher was a significant predictor for both GU toxicity and ED. To clarify the size of the relative risk of developing grade ≥ 2 GU toxicity or ED, Fig. 2 shows predicted probabilities at various levels of these risk factors.

**Table 3:** Association of pre-treatment characteristics with grade ≥ 2 toxicity.

Domain		OR	95% CI		p-value
			lower	upper	
GU	Baseline toxicity	14.76	6.14	35.50	**<0.01**
	Baseline IPSS	1.11	1.01	1.23	**0.03**
	Urethra D10% (Gy)	1.28	1.05	1.56	**0.01**
	Bladder D1cc (Gy)	1.02	0.84	1.24	0.85
	Prostate size (cc)	1.02	0.98	1.05	0.36
	Tumor stage > T2	1.10	0.29	4.20	0.89
	Size of the CTV (cc)	1.04	0.89	1.21	0.61
	Primary LDR-BT (versus EBRT)	2.79	0.90	8.70	0.08
	Interval primary–salvage treatment (years)	1.07	0.95	1.22	0.26
	Previous salvage treatment	0.72	0.05	11.31	0.81
	History of TURP	2.46	0.34	17.95	0.37
	Number of brachytherapy catheters used	0.83	0.59	1.16	0.28

ED	Baseline toxicity	73.70	15.97	340.03	**<0.01**
	Baseline IIEF	0.97	0.87	1.08	0.56
	Dorsolateral location of the tumor	2.81	0.25	31.19	0.40
	Prostate size (cc)	1.00	0.95	1.05	0.96
	Tumor stage > T2	0.45	0.07	2.76	0.39
	Size of the CTV (cc)	1.04	0.85	1.27	0.70
	Primary LDR-BT (versus EBRT)	1.73	0.28	10.62	0.55
	Interval primary–salvage treatment (years)	1.10	0.88	1.37	0.40
	Previous salvage treatment	0.31	0.01	12.01	0.53
	Previous use of ADT*	1.58	0.17	15.08	0.69
	Number of brachytherapy catheters used	1.04	0.64	1.68	0.88

Legend: GU: genitourinary, ED: erectile dysfunction, IPSS: international prostate symptoms score, CTV: clinical tumor volume, LDR-BT: low-dose-rate brachytherapy, EBRT: external beam radiotherapy, TURP: transurethral resection of the prostate, IIEF: international index of erectile function, ADT: androgen deprivation therapy, OR: odds ratio, 95% CI: 95% confidence interval.

* As part of primary treatment (neo-adjuvant or adjuvant).

## 4 Discussion

For patients with a local prostate cancer recurrence after radiotherapy, the tradeoff between tumor control and risk of normal tissue damage needs close evaluation when offering salvage treatment. This study provides a comprehensive insight into the occurrence of toxicity after ultrafocal salvage HDR-BT. While severe (grade 3) toxicity was very low (3% GU, 0% GI), proving the safety of this treatment, the number of patients experiencing mild (grade 1) or moderate (grade 2) toxicity was more pronounced (GU 32% and 41%, GI 31% and 5%, respectively). Although almost half of all patients already had grade ≥ 2 ED at baseline, new grade 2 and 3 ED was seen in 22% and 15%, respectively.

A further evaluation of individual toxicity subdomains and timing of occurrence shows that there are certain patterns of toxicity over time. The acute phase after treatment was mainly characterized by transient mild symptoms of haematuria, urinary frequency and urinary tract pain, which are common acute symptoms after brachytherapy. In the late phase, moderate urinary frequency became more frequent, as well as moderate urinary incontinence and urinary retention. Erectile function generally decreased over time, with increasing frequencies across the range of mild to severe symptoms.

Although the CTCAE is commonly used to describe treatment-related toxicities, the severity of symptoms and their grading varies between subdomains. The general guideline states that grade 2 toxicity refers to moderate symptoms indicating minimal, local or noninvasive intervention, whereas grade 3 toxicity involves disabling symptoms limiting self-care activities of daily living or (prolongation of) hospitalization [12]. Within the subdomain urinary retention, grade 2 toxicity includes placement of a urinary or suprapubic catheter or intermittent catheterization, besides use of medication. In our group, 7 patients with grade 2 urinary retention required a (temporary) urinary catheter (2/7), a (temporary) suprapubic catheter (4/7) or needed self-catheterization (1/7). Since these interventions have substantial impact on daily life activities, we urge to report them separately.

In recent years, an increasing amount of literature on focal salvage HDR-BT has become available. Table 4 summarizes four studies using different focal HDR-BT regimens and targeting strategies, who all reported GU and GI toxicities using the CTCAE 4.0 [13], [14], [15], [16]. Across these studies, 2–10% grade 3 GU and 0% grade 3 GI toxicity was reported. Acute and late grade 2 GU toxicity was observed in 54–93% and 42–47% of patients. Two studies specified grade 2 retention: Murgic et al. described that no patient required a urinary catheter, while Chitmanee et al. had patients requiring intermittent catheterization (n = 9), urethral dilatation (n = 1) and a suprapubic catheter (n = 1). Acute and late grade 2 GI toxicity occurred in 0–8% and 0–13%. Grade 1 GU toxicity was observed in 0–36% (acute) and 20–26% (late), and grade 1 GI toxicity in 14–24% (acute) and 14–22% (late).

**Table 4:** Focal salvage HDR-BT studies reporting CTCAE 4.0 toxicity.

Study	Year	N	Dose regimen	Target	OAR dose constraints	Median follow-up	Grade 3 toxicity
Jiang et al (13)	2017	22	3 weekly fractions of 10 Gy	Peripheral zone and choline PET-positive area	≤9 Gy to the urethral surface and ≤ 7 Gy to the visible rectum	73 months	GI: 0% GU: 9% (late phase)
Murgic et al (14)	2018	15	2 weekly fractions of 13.5 Gy	Prostate quadrant with MRI-visible lesion	Urethra D10% <110% and rectal V80% <0.2 ml	36 months	GI: 0% GU: 6.7% (late phase)
Slevin et al (15)	2020	43	Single dose of 19 Gy	Lesion as identified using TRUS, mp-MRI, PET-CT and template-guided biopsies	Urethra D10% <20.9 Gy, rectal V100%=0 ml and rectal D2cc < 12.35 Gy	26 months	GI: 0% GU: 2.3% (late phase)
Chitmanee et al (16)	2020	50	Single dose of 19 Gy	Lesion as identified using mp-MRI and template mapping biopsies	Urethra D10% <22 Gy and rectal D2cc < 15 Gy	21 months	GI: 0% GU: 10% (late phase)

Legend: CTCAE: Common Terminology Criteria for Adverse Events, N: number of patients, OAR: organs at risk, Gy: Gray, PET: positron emission tomography, mp-MRI: multiparametric magnetic resonance imaging, TRUS: transrectal ultrasound, GU: genitourinary, GI: gastro-intestinal.

In comparison, retrospective studies on other focal salvage modalities such as HIFU, cryotherapy and irreversible electroporation (IRE) have described similarly low complication rates [17], [18], [19]. However, future results from prospective multi-center trials will provide more insight in the role of focal salvage IRE (FIRE trial, ACTRN12617000806369) and focal salvage HIFU/cryotherapy (FORECAST trial, NCT01883128).

In a previous study, we focused on patient-reported quality of life after ultrafocal salvage HDR-BT [9]. Patients reported increased urinary symptoms (especially in the first month after treatment) and a decrease of sexual functioning, while bowel symptoms were negligible. The explorative risk factor analysis in that study revealed that increased baseline urinary symptoms and higher urethra D10% (≥16 Gy) were significantly associated with post-treatment urinary symptoms, and impaired sexual functioning at baseline with post-treatment erectile dysfunction. These results are consistent with the current findings, in which baseline GU/ED toxicity, IPSS ≥ 14 and urethra D10% ≥17 Gy were significant predictors for grade ≥ 2 toxicity. While these analyses highlight the importance of assessing urinary and sexual function before treatment and the need for a strict urethral dose constraint, it also shows the apparent weak relationship between toxicity and other factors such as dose to the bladder, size or stage of the tumor and number of brachytherapy catheters used for the implant. This is important information to find areas of improvement for treatment planning and patient selection, especially since (ultra)focal salvage HDR-BT is being adopted in an increasing number of centers worldwide.

Out of the five patients who experienced severe GU toxicity, only two had substantial pre-treatment urinary complaints, consisting of increased frequency (hourly urination), hesitation and mild urge. Pre-treatment IPSS values among these patients ranged between 3 and 18. A common denominator was the relatively high received dose by the urethra, with D10% >17 Gy in 4/5 patients.

Although beyond the scope of this study, more research is warranted to explore potential improvements in terms of optimizing tumor control. For instance, dose fractionation may offer biological advantages. As described above, different dosimetry and fractionation schemes are being employed for (ultra)focal salvage HDR-BT. Although toxicity seems comparable between these regimens, estimated 3-year biochemical disease-free survival was higher in the multi-fraction studies (±60%) than the single-dose studies (±44%). Recent results from a comparative trial on the efficacy of whole-gland HDR-BT in the primary setting (1 × 19 Gy versus 2 × 13.5 Gy) revealed a clear 5-year cancer control advantage for the two-fraction arm [20]. Using patient- and tumor-related characteristics, we are currently in the process of developing a prediction model for biochemical failure to further optimize our patient selection criteria.

## 5 Conclusions

MRI-guided ultrafocal salvage HDR-BT can be offered as a safe salvage treatment to patients with a local recurrence after primary radiotherapy. Adequate patient selection by baseline symptom assessment and adherence to urethral dose constraints during treatment planning are the most important factors to avoid (severe) toxicity. By offering this treatment, patients may avoid or at least postpone the need for ADT, preventing them from hormone deprivation-related symptoms. Further research in this field should focus on potential areas of improvement in terms of cancer control, aiming to maintain patients ADT-free for as long as possible.

## Disclosure statement

M.J. van Son: nothing to disclose.

M. Peters: received a grant by the Dutch Cancer Sociey (KWF Kankerbestrijding) [grant number 10932].

M.A. Moerland: nothing to disclose.

S.M.G. van de Pol: nothing to disclose.

W.S.C. Eppinga: nothing to disclose.

J.J.W. Lagendijk: nothing to disclose.

J.R.N. van der Voort van Zyp: received a grant by the Dutch Cancer Sociey (KWF Kankerbestrijding) [grant number 10932].

## Funding

This work was supported by the Dutch Cancer Sociey (KWF Kankerbestrijding) [grant number 10932]. The funding source had no involvement in study design, in the collection, analysis or interpretation of data, in the writing of the report or in the decision to submit the article for publication.

## Declaration of Competing Interest

The authors declare that they have no known competing financial interests or personal relationships that could have appeared to influence the work reported in this paper.
