# Supplementary material for: Determining the safety of ultrafocal salvage high-dose-rate brachytherapy for radiorecurrent prostate cancer: A toxicity assessment of 150 patients
Source: Clin Transl Radiat Oncol. 2020 Dec 11;27:1–7. doi: 10.1016/j.ctro.2020.12.002 (PMC7750686; doi:10.1016/j.ctro.2020.12.002)
Supplement: Supplementary data 1 [file mmc1.docx]

**Supplementary files**

Manuscript: “Toxicity assessment of ultrafocal salvage high-dose-rate brachytherapy for radiorecurrent prostate cancer.”

| **Supplementary Table 1 – New-onset acute and late toxicity per subdomain** | | | | |
| --- | --- | --- | --- | --- |
| *Domain* | *Acute*  *number (%)* | *Missing, n* | *Late*  *number (%)* | *Missing, n* |
| ***Genitourinary toxicity*** | | | | |
| Cystitis  No toxicity  Grade 1  Grade 2  Grade 3 | 140 (97.2%)  2 (1.4%)  2 (1.4%)  0 (0%) | 6 | 124 (97.6%)  1 (0.8%)  1 (0.8%)  1 (0.8%) | 23 |
| Haematuria  No toxicity  Grade 1  Grade 2  Grade 3 | 104 (72.2%)  38 (26.4%)  2 (1.4%)  0 (0%) | 6 | 119 (93.7%)  7 (5.5%)  1 (0.8%)  0 (0%) | 23 |
| Urinary frequency  No toxicity  Grade 1  Grade 2  Grade 3 | 103 (71.5%)  24 (16.7%)  17 (11.8%)  0 (0%) | 6 | 72 (56.7%)  11 (8.7%)  44 (34.6%)  0 (0%) | 23 |
| Urinary incontinence  No toxicity  Grade 1  Grade 2  Grade 3 | 120 (83.3%)  18 (12.5%)  6 (4.2%)  0 (0%) | 6 | 98 (77.1%)  17 (13.4%)  10 (7.9%)  2 (1.6%) | 23 |
| Urinary retention  No toxicity  Grade 1  Grade 2  Grade 3 | 130 (90.3%)  11 (7.6%)  3 (2.1%)  0 (0%) | 6 | 102 (80.3%)  14 (11%)  9 (7.1%)  2 (1.6%) | 23 |
| Urinary tract pain  No toxicity  Grade 1  Grade 2  Grade 3 | 95 (66%)  44 (30.5%)  5 (3.5%)  0 (0%) | 6 | 114 (89.8%)  10 (7.9%)  3 (2.3%)  0 (0%) | 23 |
| ***Gastrointestinal toxicity*** | | | | |
| Abdominal pain  No toxicity  Grade 1  Grade 2  Grade 3 | 141 (97.9%)  2 (1.4%)  1 (0.7%)  0 (0%) | 6 | 122 (96.1%)  5 (3.9%)  0 (0%)  0 (0%) | 23 |
| Diarrhoea  No toxicity  Grade 1  Grade 2  Grade 3 | 141 (97.9%)  3 (2.1%)  0 (0%)  0 (0%) | 6 | 119 (93.7%)  8 (6.3%)  0 (0%)  0 (0%) | 23 |
| Enterocolitis  No toxicity  Grade 1  Grade 2  Grade 3 | 142 (98.6%)  2 (1.4%)  0 (0%)  0 (0%) | 6 | 127 (100%)  0 (0%)  0 (0%)  0 (0%) | 23 |
| Fecal incontinence  No toxicity  Grade 1  Grade 2  Grade 3 | 140 (97.2%)  3 (2.1%)  1 (0.7%)  0 (0%) | 6 | 119 (93.7%)  7 (5.5%)  1 (0.8%)  0 (0%) | 23 |
| Flatulence  No toxicity  Grade 1  Grade 2  Grade 3 | 133 (92.4%)  11 (7.6%)  0 (0%)  0 (0%) | 6 | 119 (93.7%)  8 (6.3%)  0 (0%)  0 (0%) | 23 |
| Hemorrhoids  No toxicity  Grade 1  Grade 2  Grade 3 | 136 (94.4%)  8 (5.6%)  0 (0%)  0 (0%) | 6 | 122 (96.1%)  4 (3.1%)  1 (0.8%)  0 (0%) | 23 |
| Proctitis  No toxicity  Grade 1  Grade 2  Grade 3 | 138 (95.8%)  6 (4.2%)  0 (0%)  0 (0%) | 6 | 124 (97.6%)  3 (2.4%)  0 (0%)  0 (0%) | 23 |
| Rectal fistula  No toxicity  Grade 1  Grade 2  Grade 3 | 144 (100%)  0 (0%)  0 (0%)  0 (0%) | 6 | 127 (100%)  0 (0%)  0 (0%)  0 (0%) | 23 |
| Rectal haemorrhage  No toxicity  Grade 1  Grade 2  Grade 3 | 140 (97.2%)  4 (2.8%)  0 (0%)  0 (0%) | 6 | 117 (92.2%)  6 (4.7%)  4 (3.1%)  0 (0%) | 23 |
| Rectal pain  No toxicity  Grade 1  Grade 2  Grade 3 | 141 (97.9%)  2 (1.4%)  1 (0.7%)  0 (0%) | 6 | 125 (98.4%)  2 (1.6%)  0 (0%)  0 (0%) | 23 |
| Legend: New-onset toxicity after ultrafocal salvage HDR-BT as graded by the Common Terminology Criteria for Adverse Events (CTCAE) 4.0. Any score above baseline in the acute (≤3 months) or late (>3 months) phase was considered new-onset toxicity. | | | | |

| **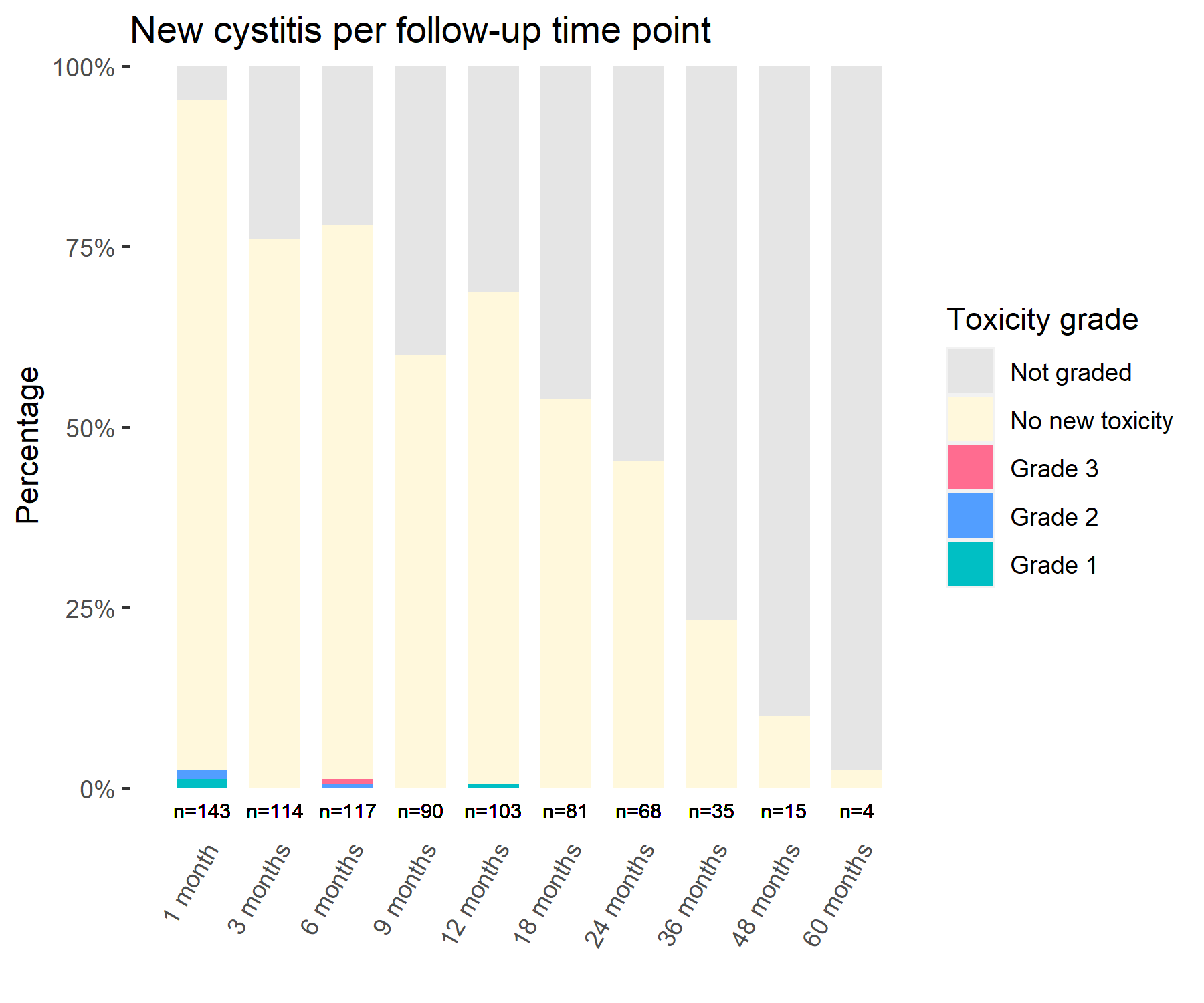** |
| --- |
| **Supplementary figure 1‑a** |
| **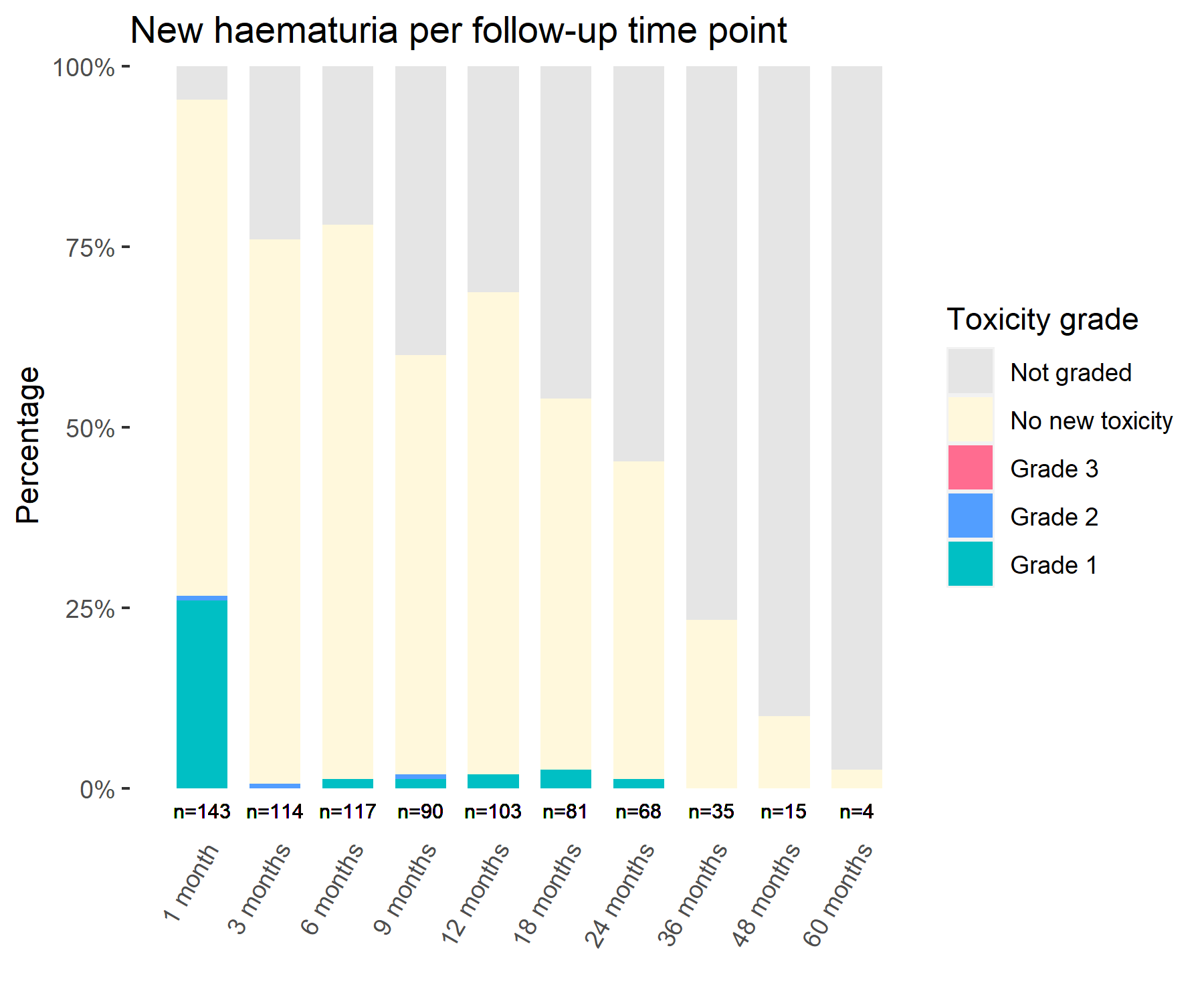** |
| **Supplementary figure 1‑b** |
| **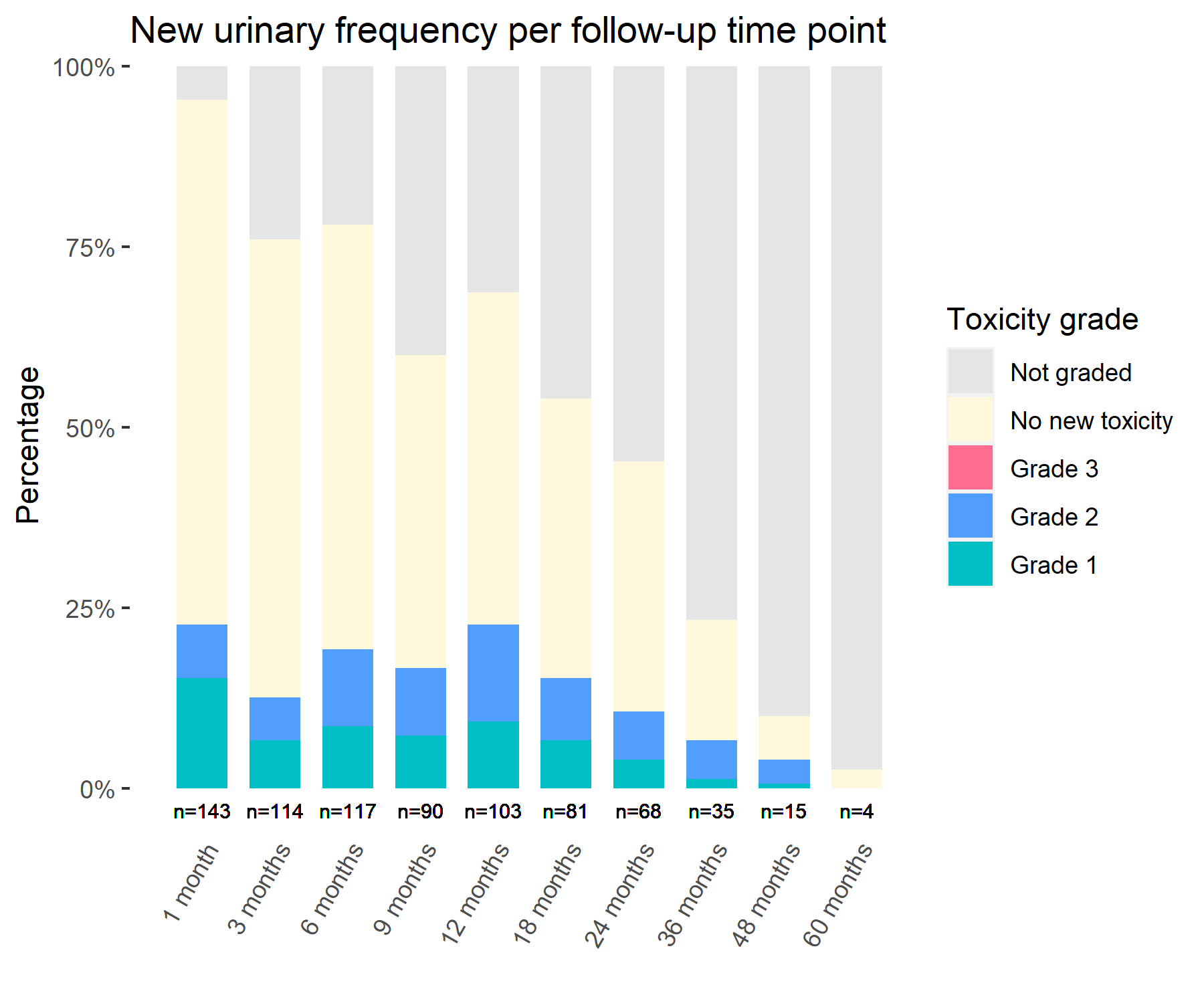** |
| **Supplementary figure 1‑c** |
| **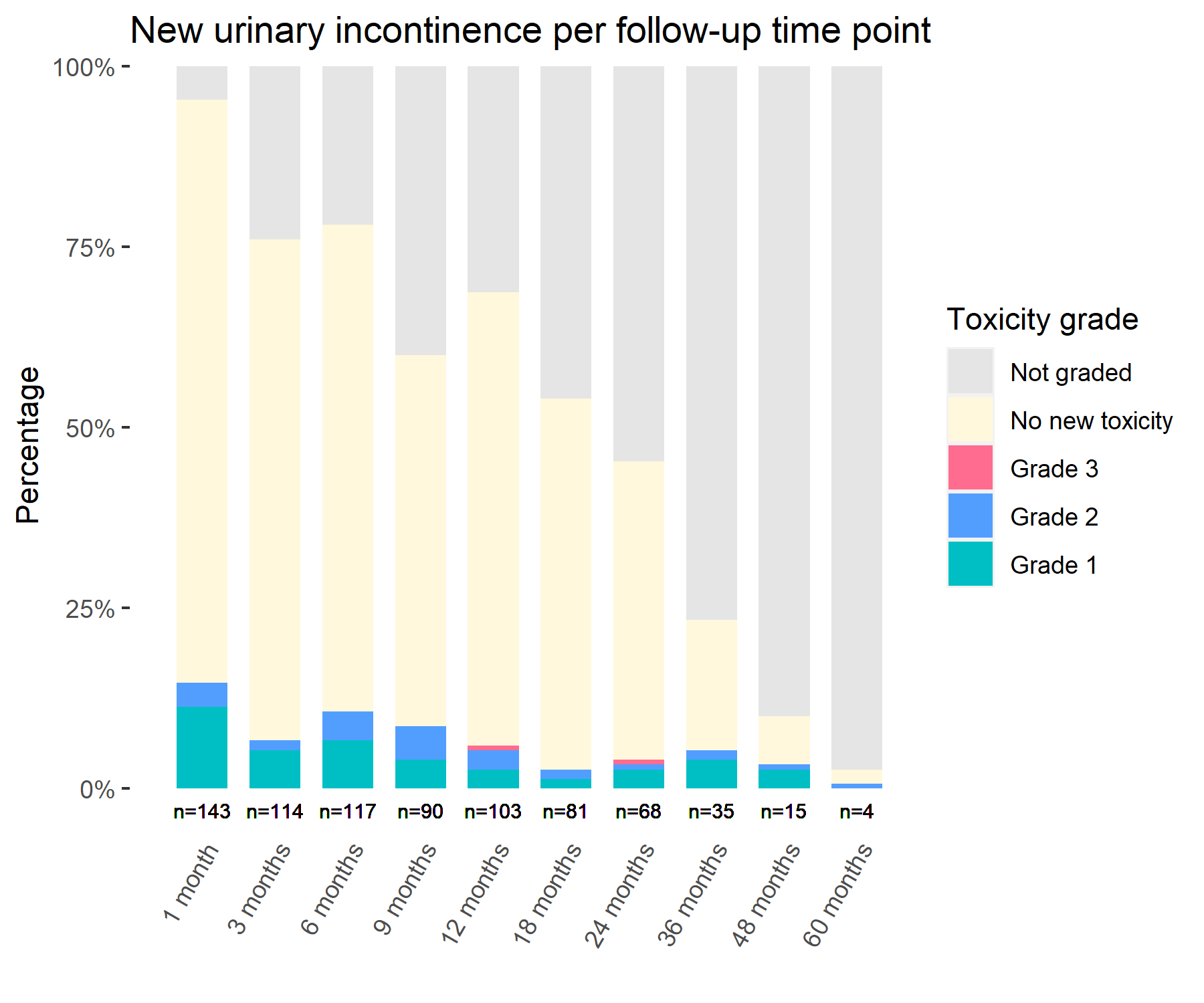** |
| **Supplementary figure 1‑d** |
| **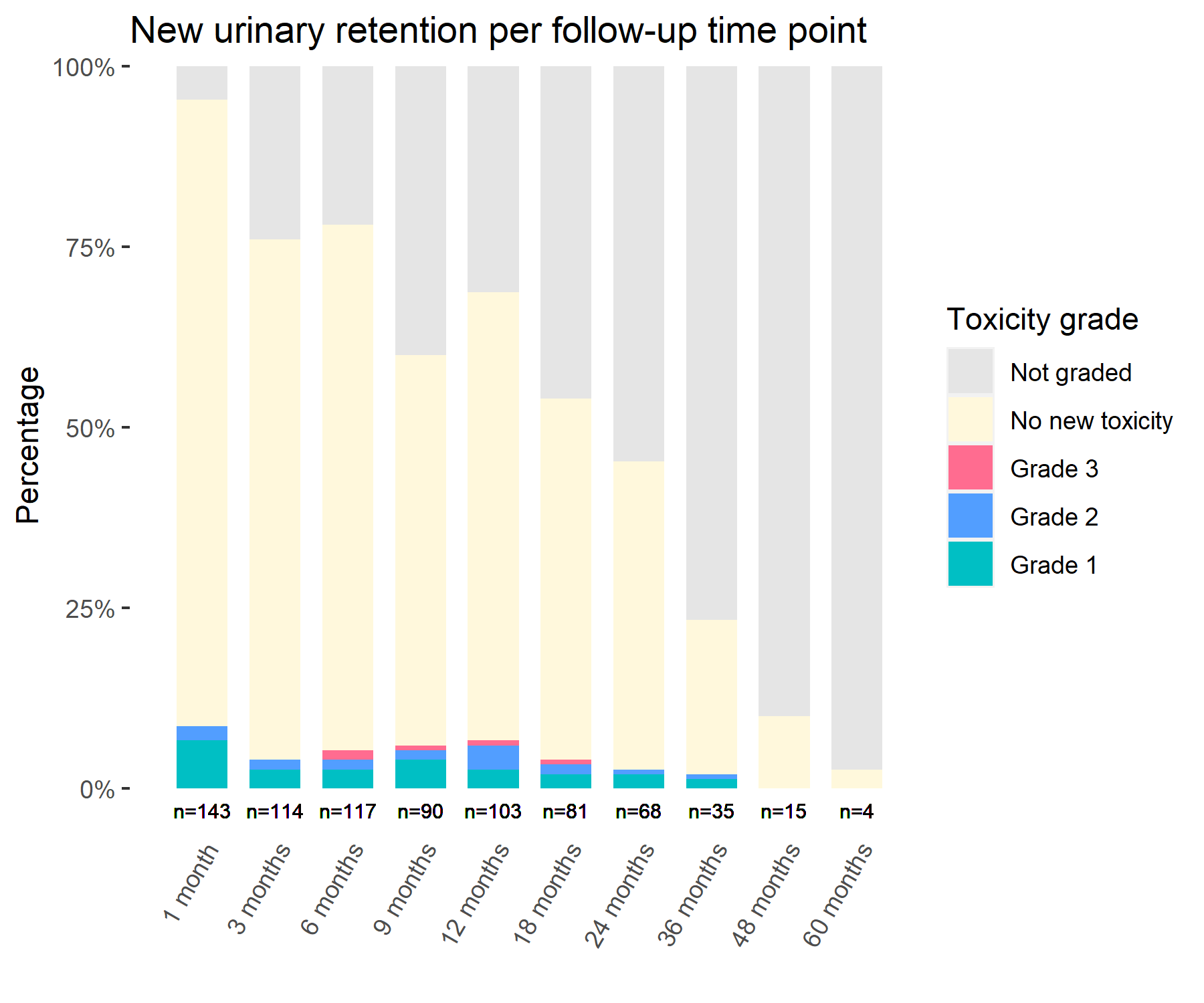** |
| **Supplementary figure 1‑e** |
| **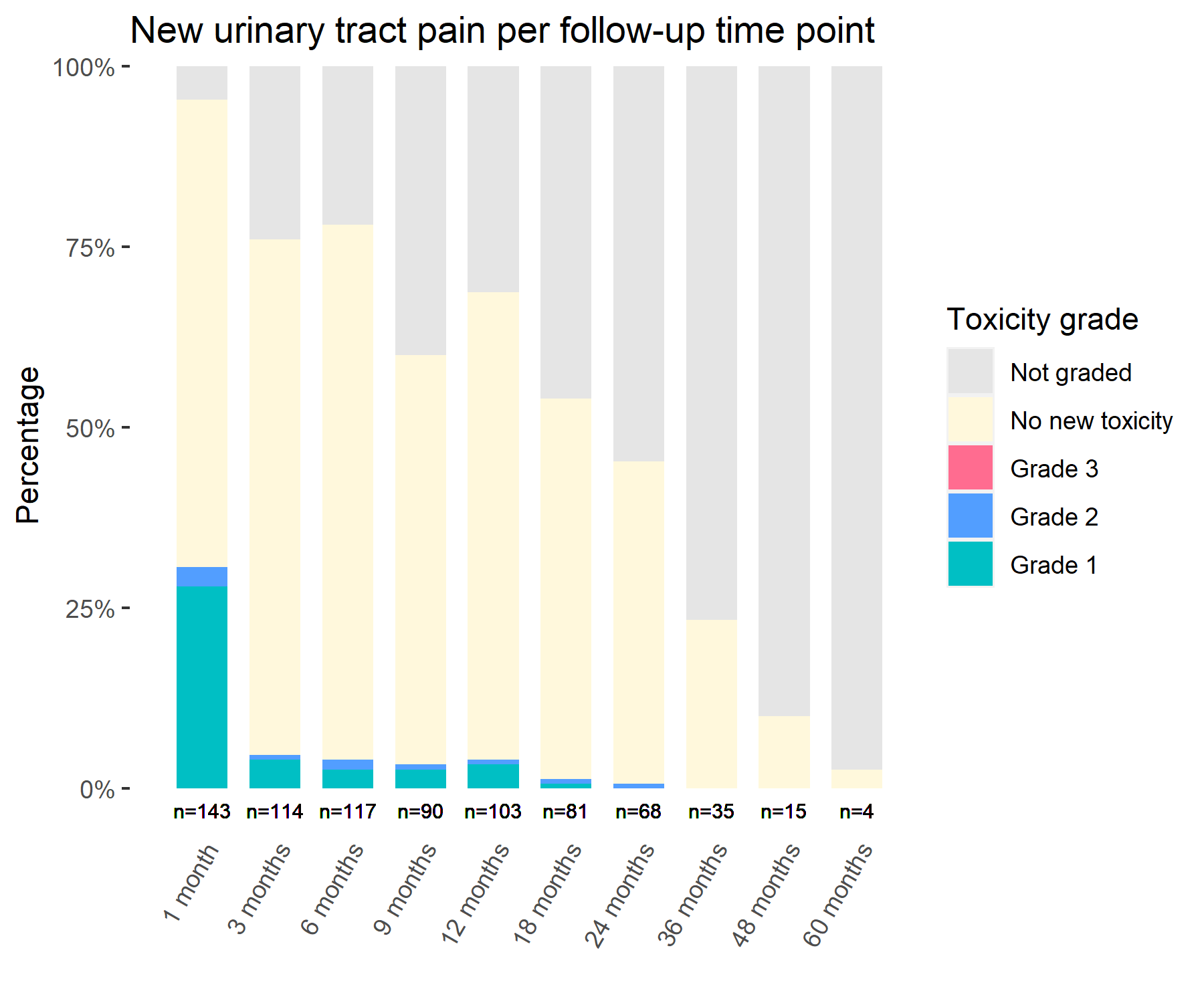** |
| **Supplementary figure 1‑f** |
| **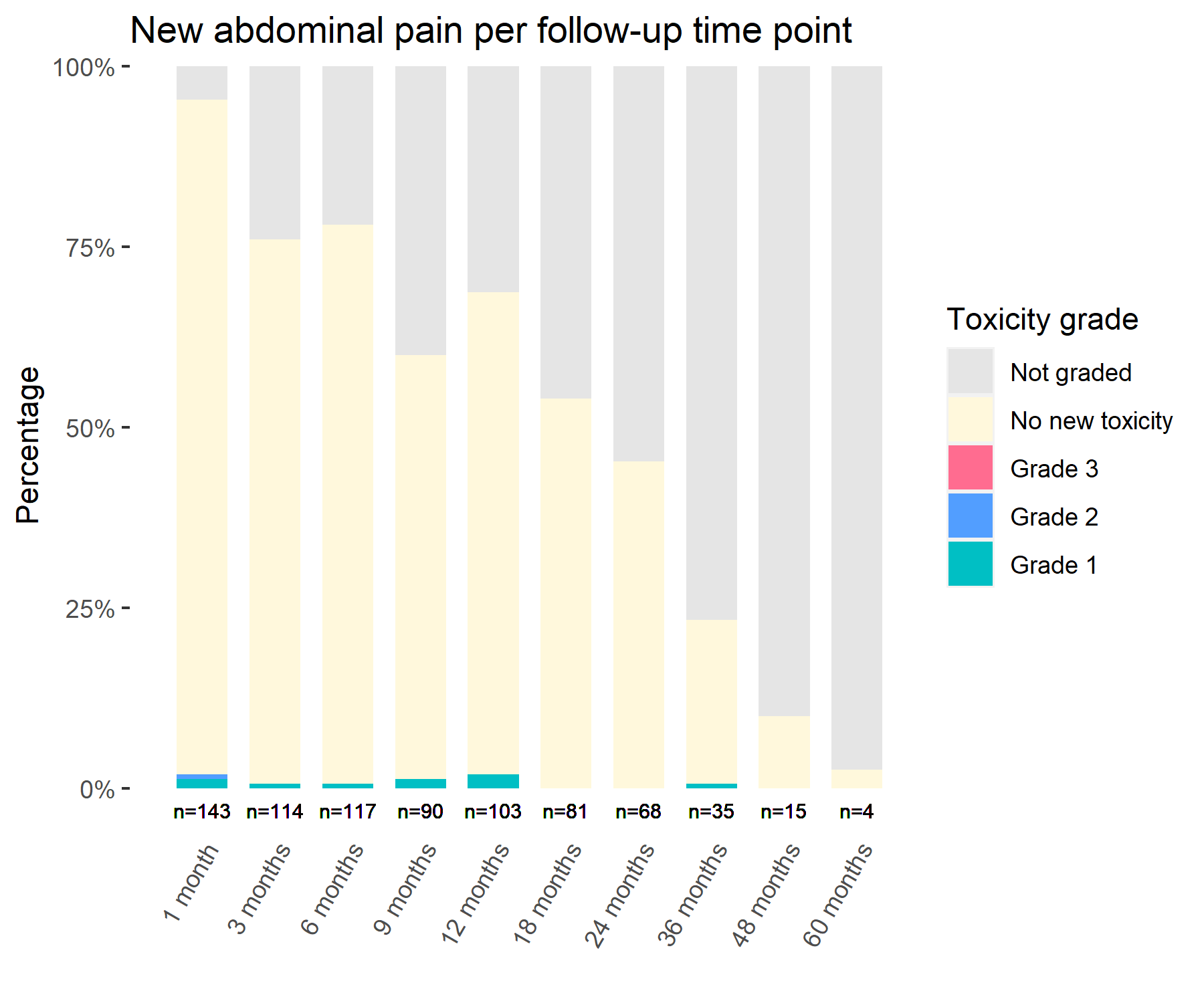** |
| **Supplementary figure 2‑a** |
| **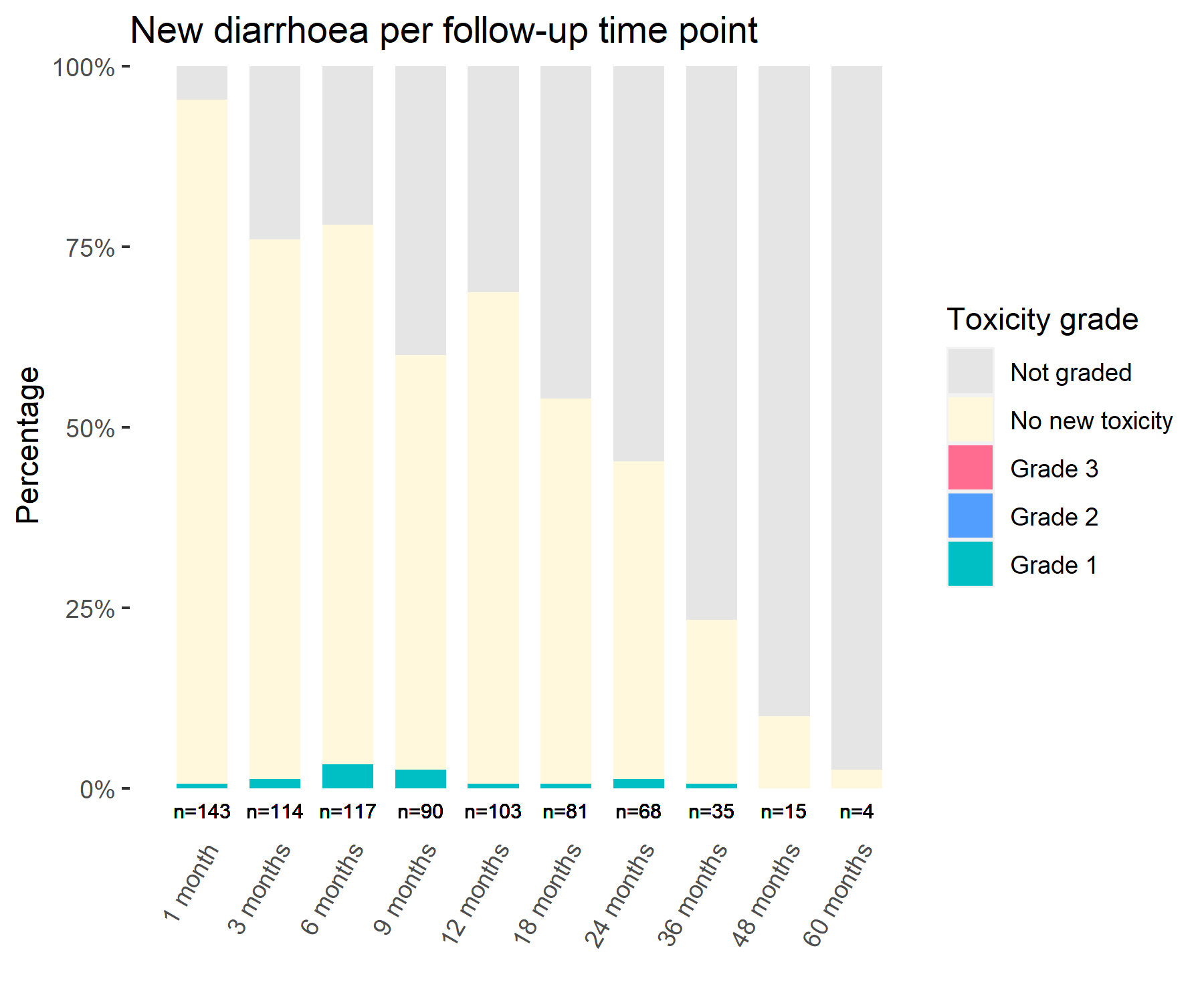** |
| **Supplementary figure 2‑b** |
| **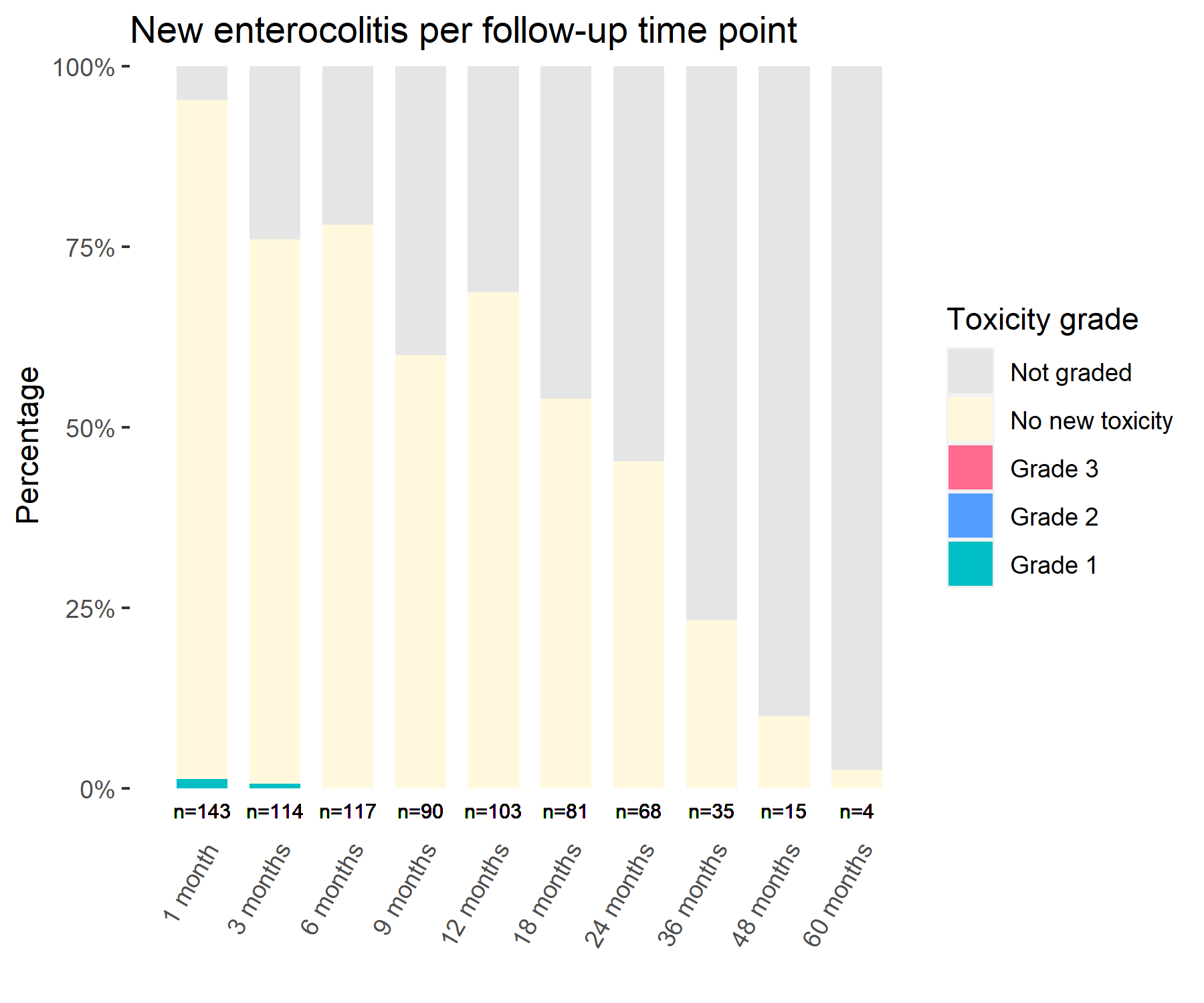** |
| **Supplementary figure 2‑c** |
| **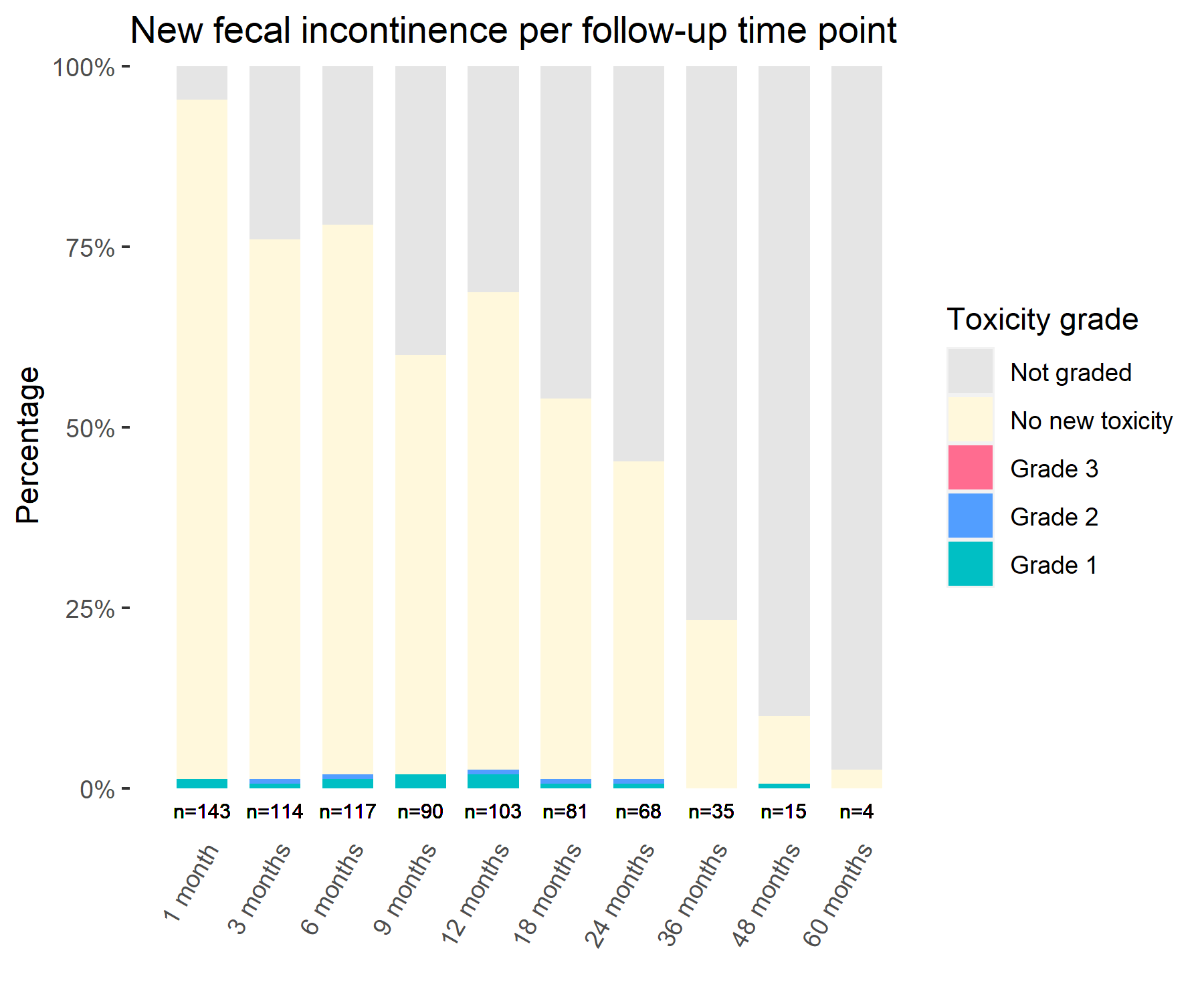** |
| **Supplementary figure 2‑d** |
| **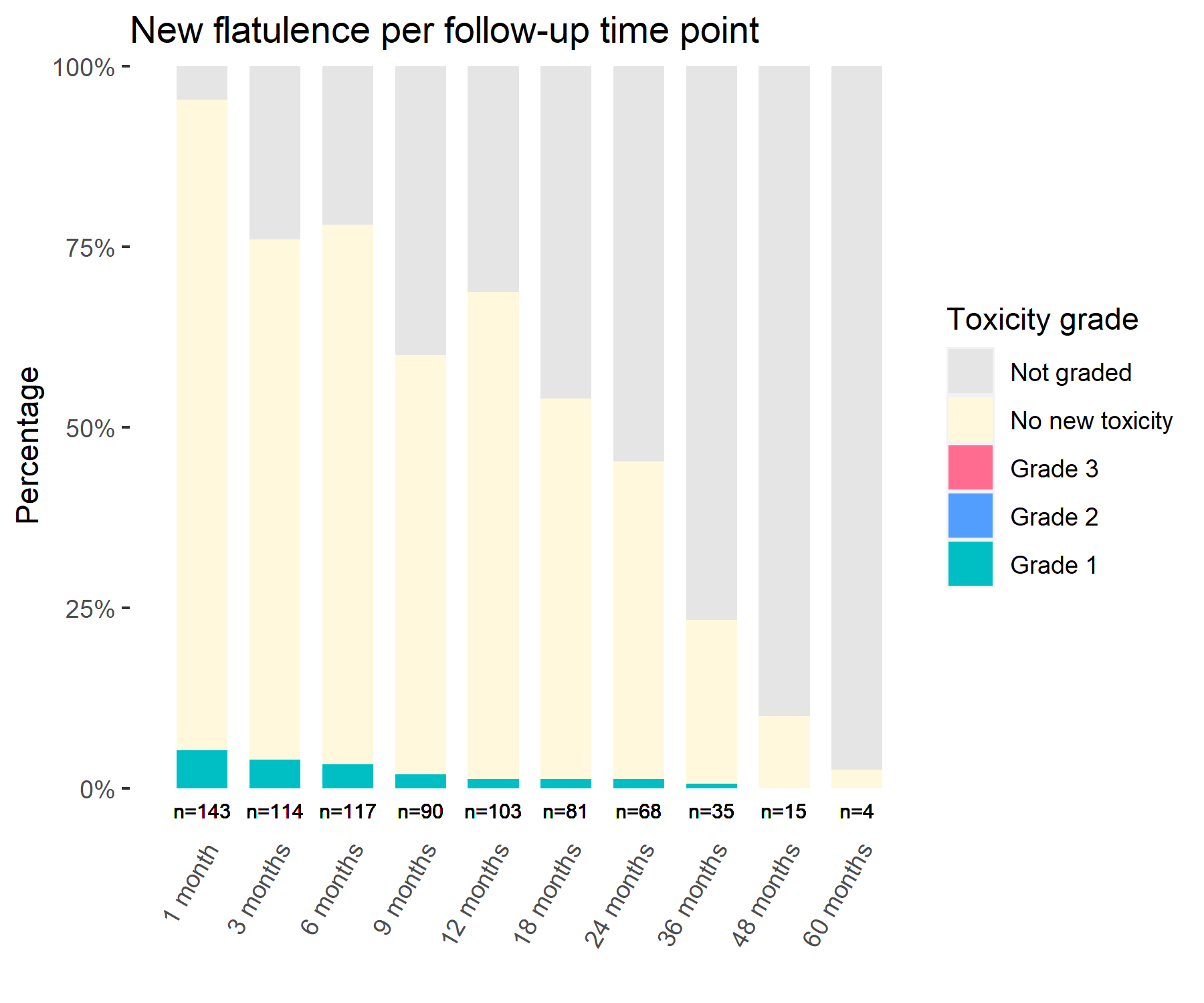** |
| **Supplementary figure 2‑e** |
| **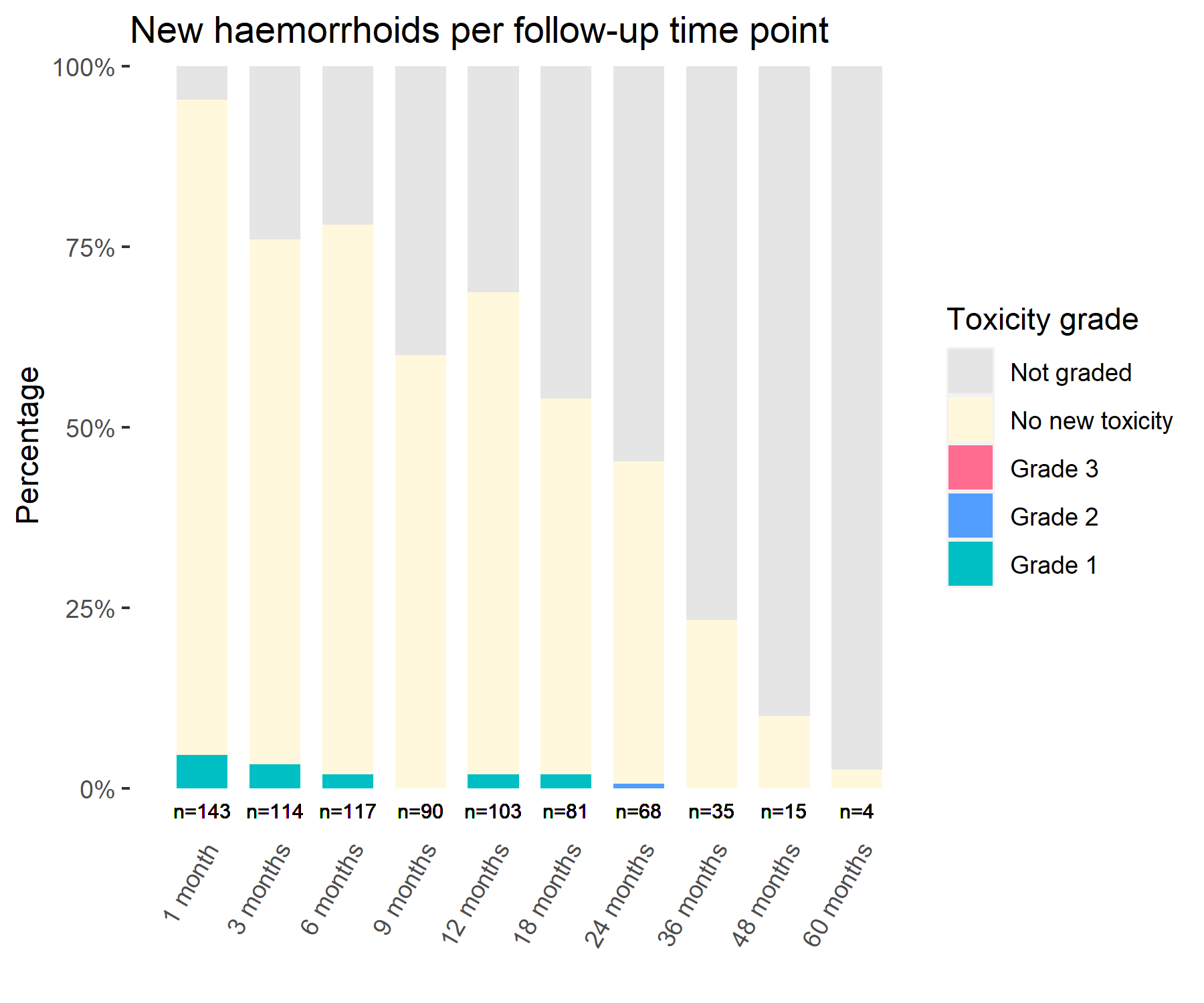** |
| **Supplementary figure 2‑f** |
| **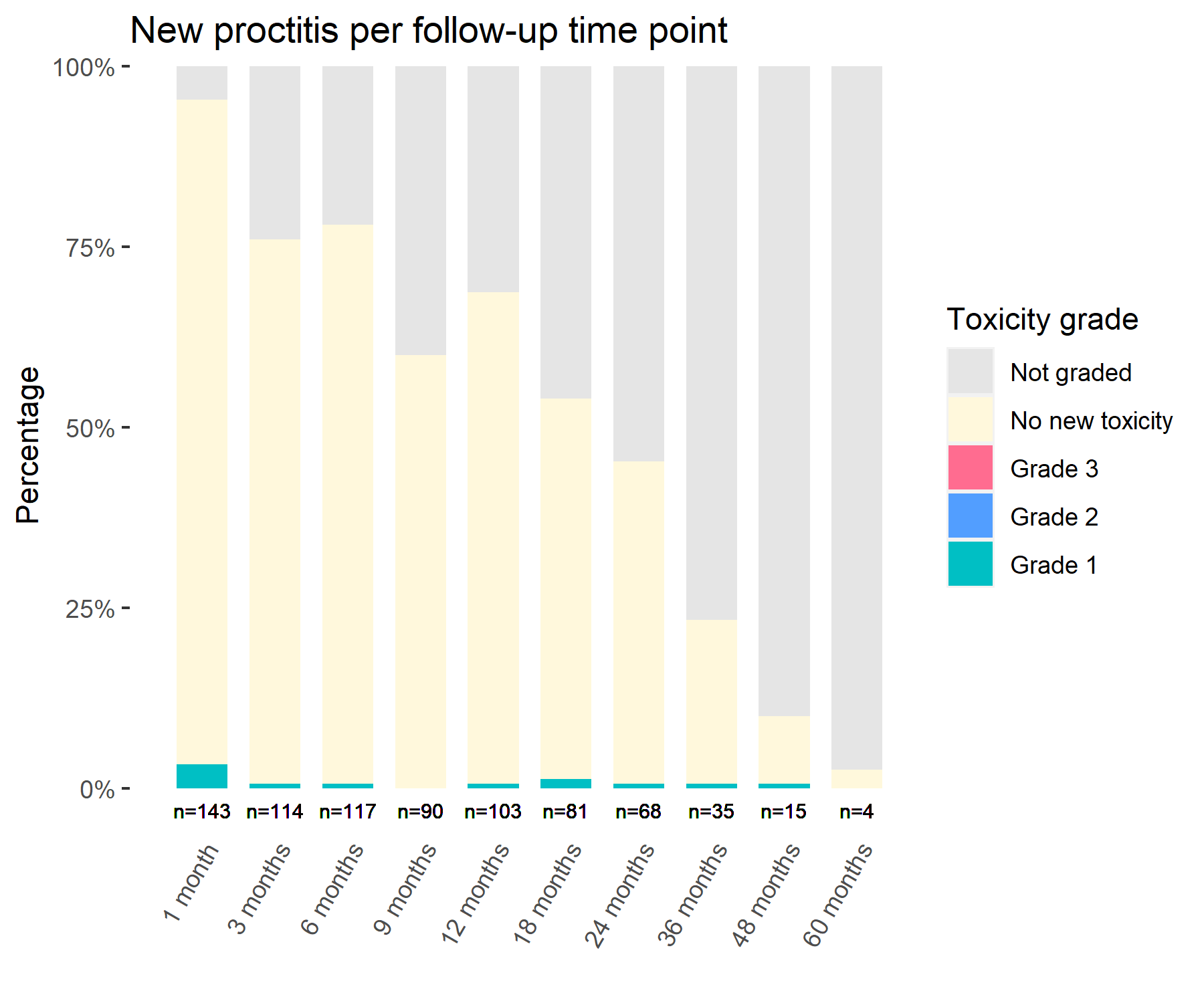** |
| **Supplementary figure 2‑g** |
| **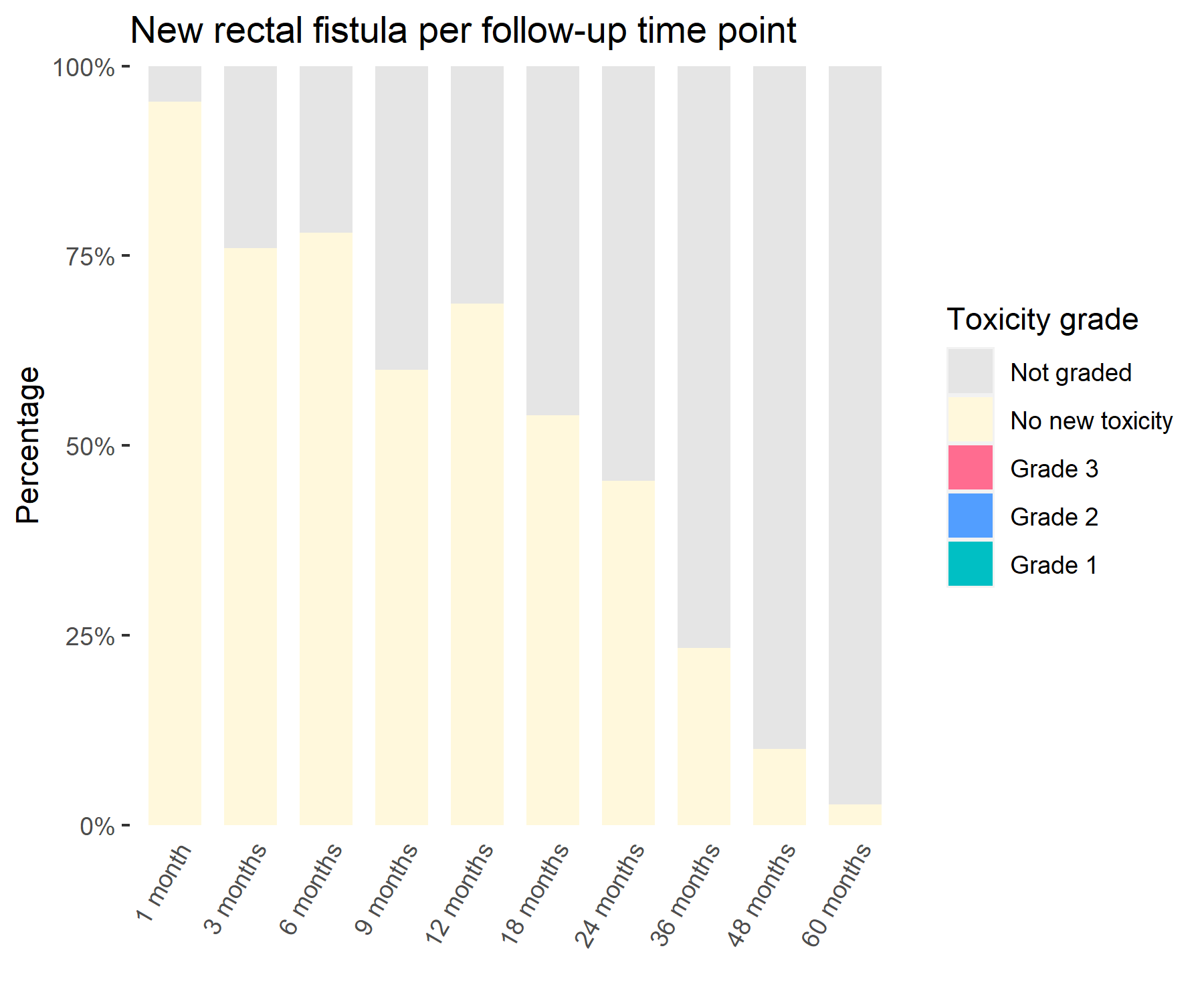** |
| **Supplementary figure 2‑h** |
| **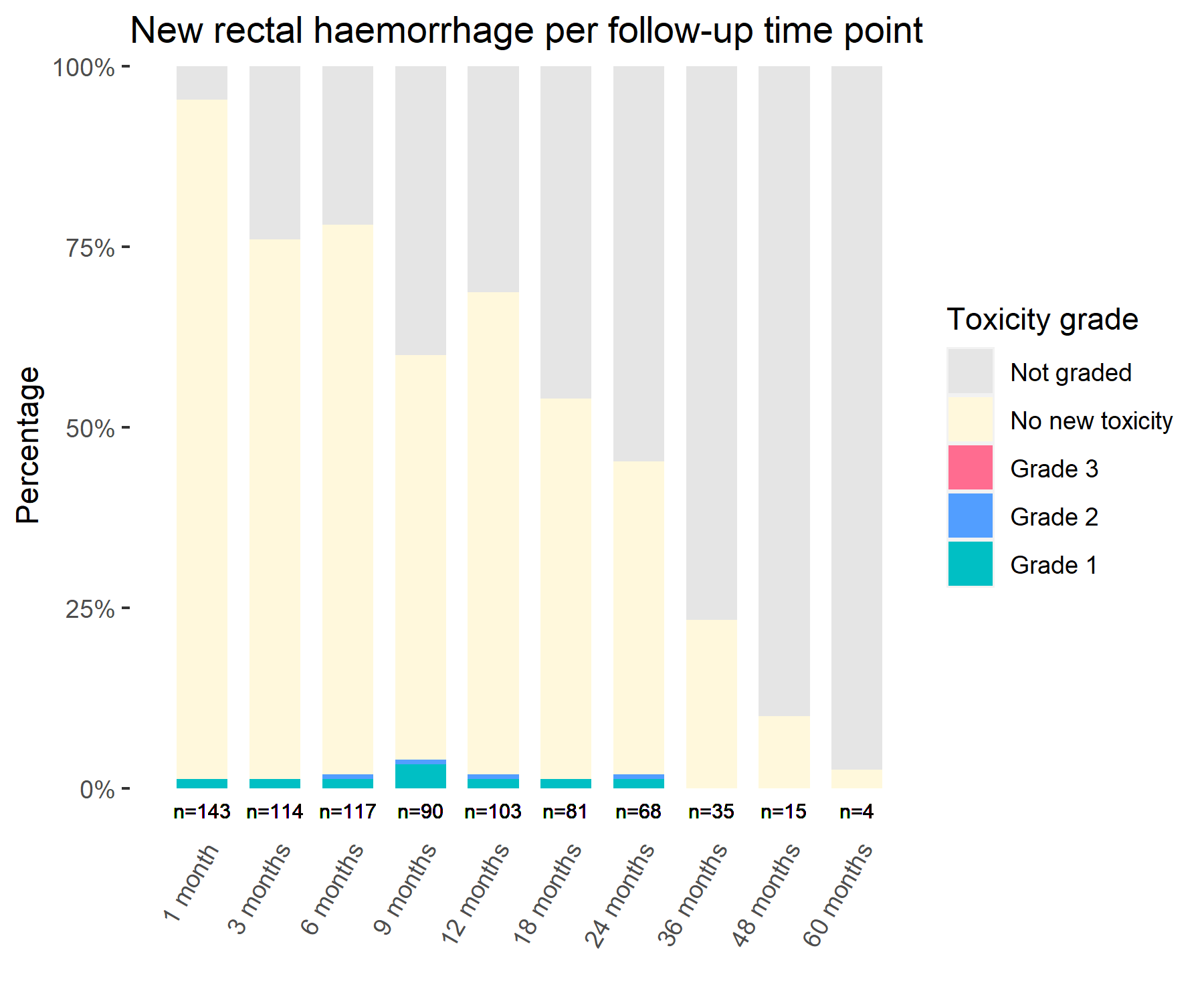** |
| **Supplementary figure 2‑i** |
| **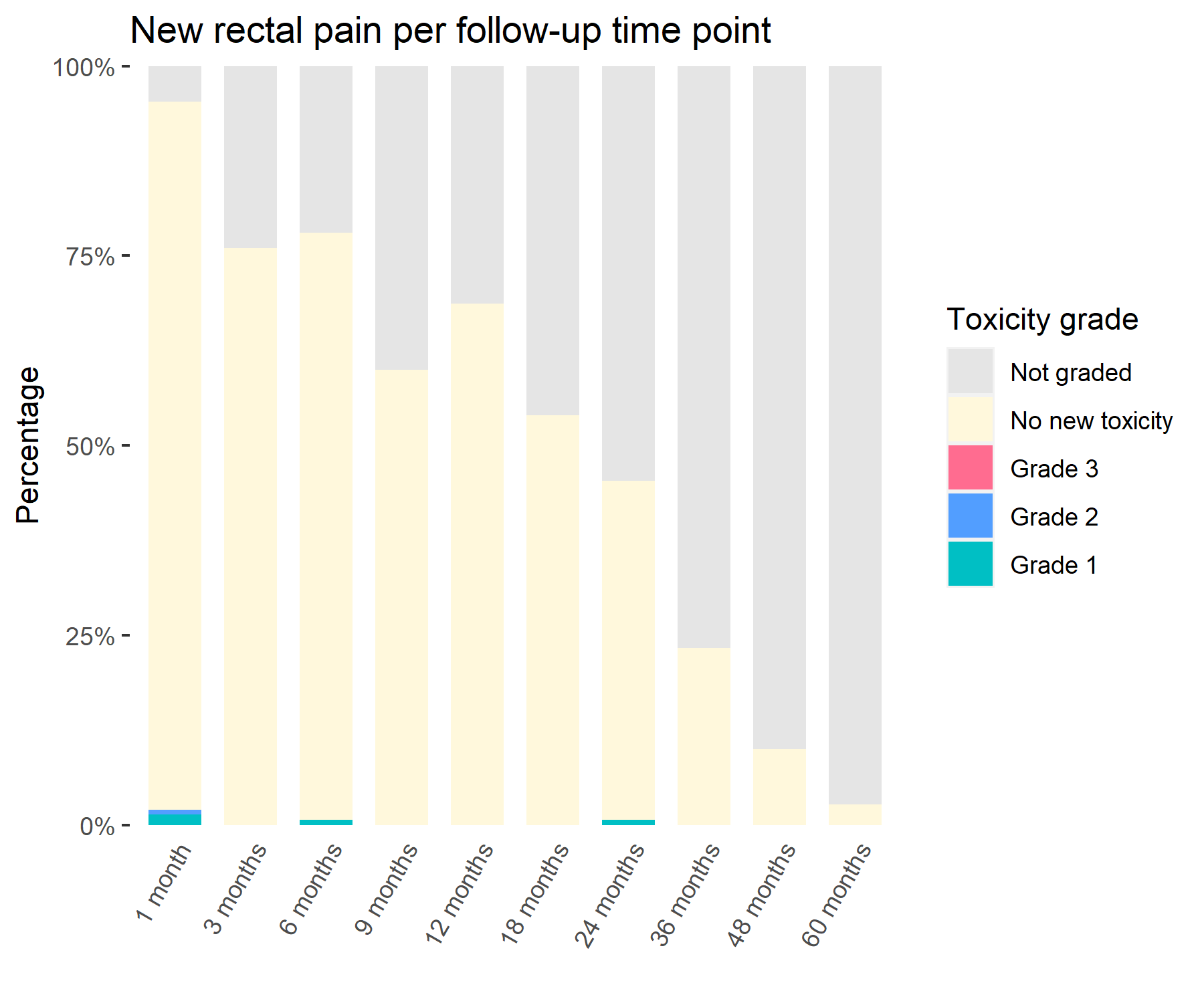** |
| **Supplementary figure 2‑j** |

**Figure captions**

**Supplementary figure 1 a–f**: Subdomains of genitourinary toxicity.

Stacked barplots displaying number of patients with new-onset toxicity after ultrafocal salvage HDR-BT. At each follow-up time point, toxicity scores were compared with baseline. Any score above baseline was considered new-onset toxicity.

**Supplementary figure 2 a–j**: Subdomains of gastro-intestinal toxicity.

Stacked barplots displaying number of patients with new-onset toxicity after ultrafocal salvage HDR-BT. At each follow-up time point, toxicity scores were compared with baseline. Any score above baseline was considered new-onset toxicity.
